# Prostatic Artery Origin Variability: Five Steps to Improve Identification during Percutaneous Embolization

**DOI:** 10.3390/medicina59122122

**Published:** 2023-12-05

**Authors:** Alexandru Șerbănoiu, Rareș Nechifor, Andreea Nicoleta Marinescu, Gheorghe Iana, Ana Magdalena Bratu, Iulia Alecsandra Sălcianu, Radu Tudor Ion, Florin Mihail Filipoiu

**Affiliations:** 1 Department of Radiology and Medical Imaging, University of Medicine and Pharmacy Carol Davila, 050474 Bucharest, Romania; alexandru.serbanoiu@drd.umfcd.ro (A.Ș.);; 2 Department of Radiology and Medical Imaging, University Emergency Hospital Bucharest, 050098 Bucharest, Romania; 3 Doctoral School of “Carol Davila”, University of Medicine and Pharmacy, 700115 Bucharest, Romania; 4 Endovascular Network Bucharest, 075100, Bucharest, Romania; 5 Department of Radiology and Medical Imaging, Colțea Hospital,030171 Bucharest, Romania

**Keywords:** prostatic arterial embolization, interventional radiology, benign prostate hyperplasia, anatomy

## Abstract

*Background and Objectives*: The purpose of the current paper is to present our study on the variability in the prostatic artery origin, discuss the less frequent origins, and present the challenges of the prostatic artery embolization (PAE) procedure, thus aiding young interventional radiologists. *Materials and Methods*: We studied the origins of the prostatic artery on digital subtraction angiography (DSA) examinations from PAE procedures on 35 male pelvises (70 hemi-pelvises). *Results*: Our study has demonstrated that the most frequent origin of the prostatic artery (PA) is the internal pudendal artery (IPA), 37.1%, followed by the anterior gluteal trunk, 27.1%, and the superior vesical artery (SVA), 21.4%. Less frequent origins are the obturator artery (OBT), 11.4%, and the inferior gluteal artery (IGA), 2.8%. *Conclusions*: Compared to other studies, we notice some differences in the statistical results, but the most frequent origins remain the same. What is more important for young interventional radiologists is to be aware of all the possible origins of the PA in order to be able to offer a proper treatment to their patients. The important aspect that will ensure the success of the procedure without post-procedural complications is represented by the successful embolization of the targeted prostatic parenchyma.

## 1. Introduction

Benign prostate hyperplasia (BPH) has been a very common pathology in the male population over the past 50 years (50% incidence) associated with lower urinary tract symptoms (LUTSs) that affect the quality of life [1]. Treatment options for BPH include medication, surgery (transurethral incision of prostate, TUIP, and transurethral resection of the prostate, TURP), and minimally invasive treatments, like prostatic artery embolization (PAE) and prostatic urethral lift. Prostatic artery embolization is a minimally invasive treatment for BPH that consists of the supra-selective embolization of prostatic arteries (PAs), which reduces the volume of the prostate parenchyma and improves the symptomatology of the patients. Described in 2010 as a therapy for BPH by Carnevale, the procedure has been adapted and developed by several interventional radiology centers [2]. 

Post-procedural outcomes for PAE and TURP are similar regarding LUTSs; however, PAE has advantages such as preservation of the urinary and sexual functions. Studies on the TURP procedure note adverse reactions such as bleeding, infection, and urethral stricture, as well as increased morbidity with retrograde ejaculation and urinary incontinence [3]. Other important advantages of PAE are the minimally invasive nature of the procedure with no need for hospitalization or general anesthesia and the increased quality of life; nevertheless, incidents such as non-targeted embolization (NTE) of the bladder or rectum causing pelvic pain or rectal bleeding can occur; usually, the area of ischemia is small and the adverse reactions are transient and do not need treatment [4,5]. Penile ischemia is possible in the case of NTE due to the vascularization origin from the IIA branches, but at the same time, erectile dysfunction after PAE has not been reported [6]. The high success rate of prostatic artery embolization procedures and the decrease rate of post-procedural incidents is directly related to good knowledge of the prostatic vascularization and its variability [4].

The indication for PAE is considered to be patients with moderate to severe lower urinary tract symptoms in direct relation to a benign prostatic obstruction who do not respond to medication or have a severe adverse reaction to it [7,8].

The main purpose of this paper is to present the procedural protocol in our department and the results of the study on the arterial origin of the PA. We expect this study to serve as a guide for young interventional radiologists who want to perform PAE by presenting the protocol of the procedure from our department and the main challenges we have encountered [9]. 

## 2. Materials and Methods

This retrospective study is based on digital subtraction angiography (DSA) examinations of 35 male patients, aged 50–83 with BPH that undergo PAE. The procedures were performed from March 2021 to May 2023 by two interventional radiologists in the Endovascular Network department. All patients have agreed to participate in this study by signing the preprocedural consent and information form.

In our department for PAE, we used the left brachial approach with a 5F introducer (Terumo™, West Collins Avenue Lakewood, CO, USA) with a 5F JR (125 cm) shape catheter (Merit Medical™, South Jordan, UT, USA), a Radiofocus™ guide wire 0.035″ (180 cm) from Terumo™, and a nonionic contrast agent (Omnipaque™ 350 mL l/mL GE Healthcare™, Chicago, IL, USA). For the supra-selective catheterization of prostatic arteries, we used a Direxion Transend 14™ and pre-loaded torqueable microcatheter from Boston Scientific™ (Marlborough, MA, USA). 

We used five steps in the arterial mapping in order to identify the PA and successfully obtain the supra-selective catheterization:

1. Contralateral 45° with 10° cranio-caudal-oriented DSA in order to catheterize the internal iliac artery.
2. Ipsilateral 45° with 10° cranio-caudal-oriented DSA in order to obtain the mapping of the main branches of the iliac artery and include them in one of the Yamaki classifications (Table 1) [10];
3. After we identified the four main branches (superior gluteal artery, inferior gluteal artery, obturator artery, internal pudendal artery), we searched for the PA artery, which usually has a characteristic corkscrew aspect and parallel trajectory with the IPA.
4. The next step is represented by the positioning of the JR catheter in an optimal position near the origin of the PA and catheterization with the Direxion™ microcatheter.
5. Study the aspect of the PA: we can find two branches of the PA, which are represented by the posterior PA (vascularization of the prostatic capsule) and anterior PA (vascularization of the prostatic parenchyma) [2].

This five-step method of mapping the arterial vascularization of the pelvis has the purpose of offering an organized search of the PA and reducing the exposure to radiation while concluding in a proper prostatic embolization.

**Figure 1:**
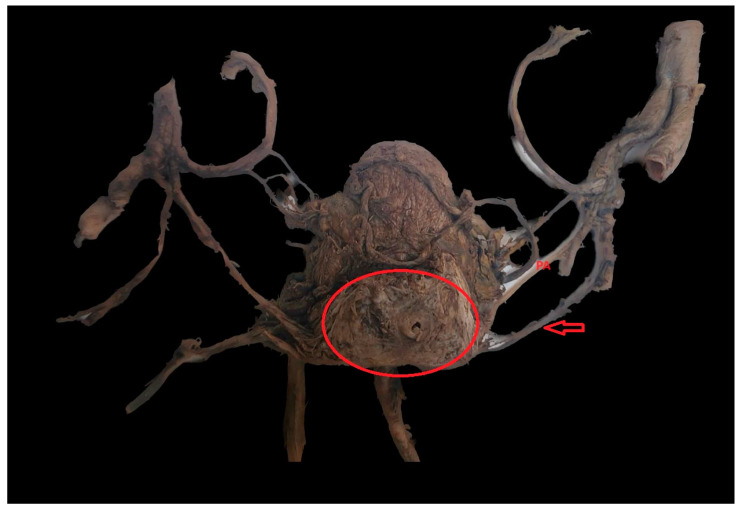
Prostate (red circle) and bladder complex obtained from anatomical dissection also with main branches from the IIA and anatomical variation with two PAs on the left side. Prostatic artery noted with PA and accessory prostate artery (arrow).

**Figure 2:**
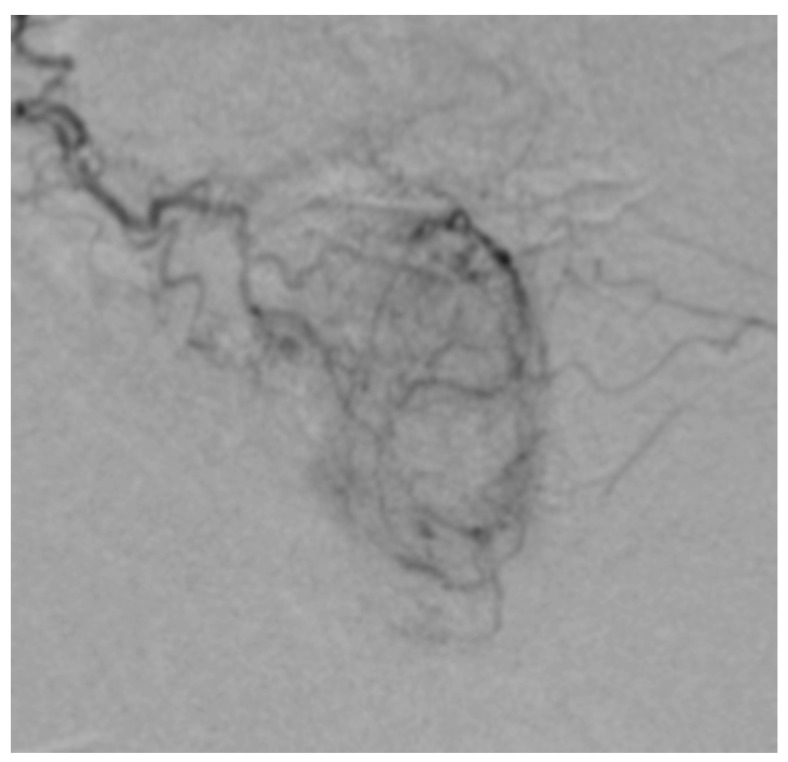
Contrast enhancement at the level of right prostatic lobe with the characteristic “blush” aspect, and we also can see the “cork-screw” aspect of the right PA.

Variabilities that must be taken into consideration when identifying the PA:

- Type of internal iliac artery (IIA) branching pattern from Yamaki classification which helps to identify possible origins of the PA [3].
- Prostatic arteries realize the vascularization of the prostate, the anterior for the prostatic parenchyma and the posterior prostatic arteries for the prostatic capsule. Depending on the variability, we can frequently find a common trunk and a distal bifurcation near the prostate, but there are also cases of two PAs with different origins (Figure 1) [11].
- There are cases when we can identify IPA and IPA accessories, which must not be confused with the PA. At the level of accessory IPAs, we can find the origin of the middle rectal artery, which has a characteristic pattern of enhancement that cannot be mistaken with the prostatic blush [12] (Figure 2);

## 3. Results

We were able to identify the origin of the PA of all patients included in this study with the most common origin found at the level of IPA (37.1% of 70 hemi-pelvises) followed by the anterior gluteal trunk above the bifurcation (27.1% of 70 hemi-pelvises), the superior vesical artery (21.4% of 70 hemi-pelvises), and the obturator artery (11.4% of 70 hemi-pelvises). Less frequent origins were found at the level of the inferior gluteal artery (2.8% of 70 hemi-pelvises). Compared with other studies, the percentage of each origin is slightly different but the main order of frequency is maintained [13,14,15] (Table 2). 

### 3.1. Prostatic Artery Origin

#### 3.1.1. PA with Origin from IPA

**Figure 3:**
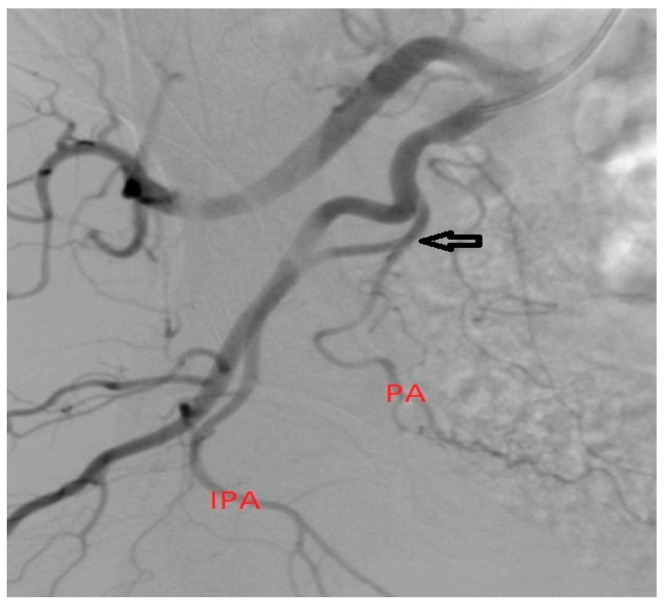
Case of a patient with PA origin (arrow) from the IPA with no significant angulation of the origin and no important collaterals.

We can consider this example as the ideal patient for PA catheterization from the point of view of the vascular anatomy. Nevertheless, it is mandatory to respect all the steps of the procedure and verify the catheterized artery before embolization(Figure 3). 

#### 3.1.2. PA with Origin from Anterior Gluteal Trunk

**Figure 4:**
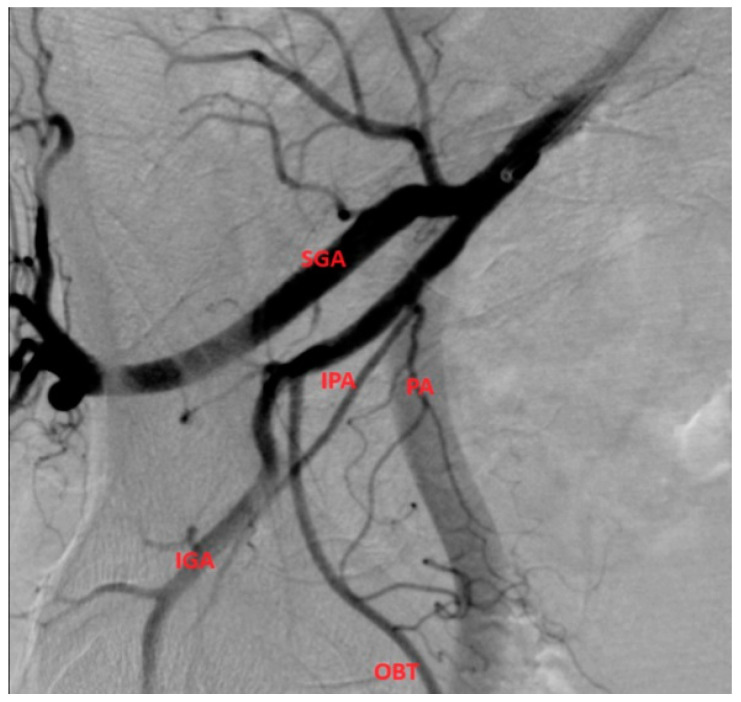
PA with origin from the anterior gluteal trunk. We can identify the obturator artery (OBT) and IPA as branches from the anterior trunk. After the origin of the PA, there are small visible arterial branches that represent inferior vesical arteries.

The origin at this level is usually accessible for catheterization with the JR catheter due to its particular shape, as it can be placed near the origin of the PA, and it will offer good support and good handling for the Direxion microcatheter (Figure 4).

#### 3.1.3. PA with Origin from SVA

**Figure 5:**
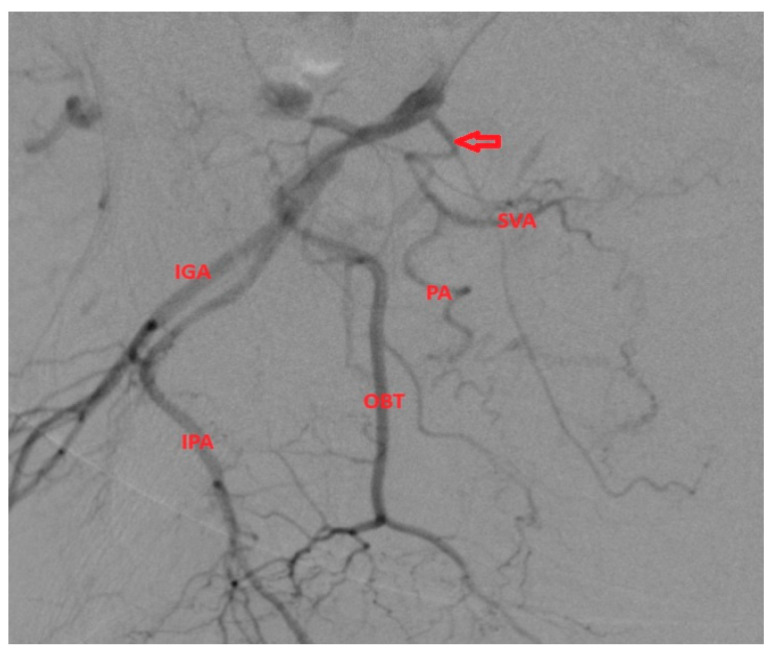
This is the case of PA with an origin from the superior vesical artery (arrow). PA has its origin in the proximal part of SVA.

For this anatomic variant, it is important to place the microcatheter distally to the PA origin in order to avoid non-targeted embolization of the bladder (Figure 5).

#### 3.1.4. PA with Distal Origin from Internal Pudendal Artery

**Figure 6:**
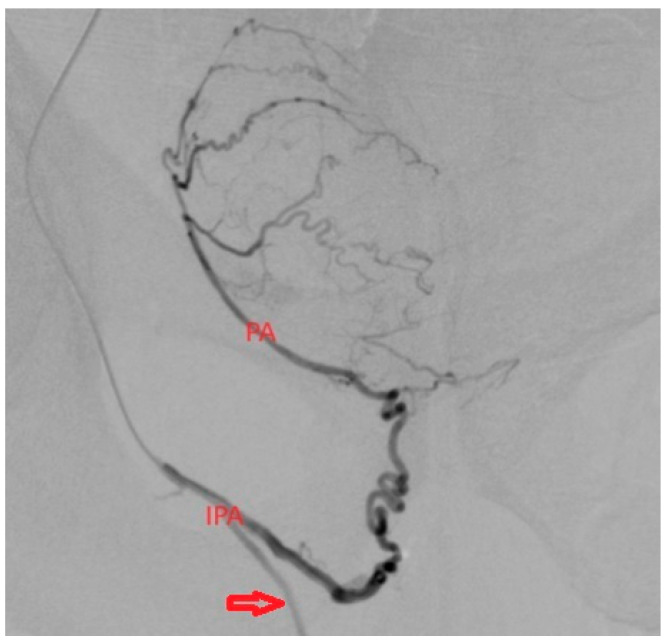
In this figure, we have a particular case of PA originating distally from the IPA with ascendent trajectory. The important aspect in this case is the supra-selective catheterization in order to not accidentally embolize the penile artery (arrow).

The most frequent origin of PA is from IPA, nevertheless distal origin is very rare and may be challenging to catheterize due to its long path and direction of the vessel (Figure 6). 

### 3.2. Importance of Supra-Selective Catheterization in Case of PA with Common Trunk or Anastomosis

#### 3.2.1. PA origin with Common Trunk

**Figure 7:**
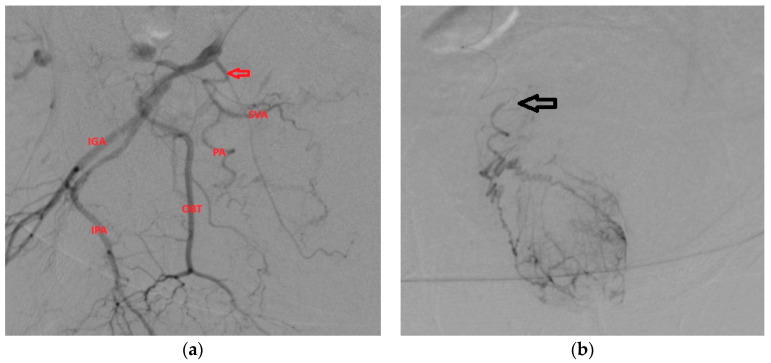
(**a**) Origin of PA from SVA (arrow); (**b**) correct position of microcatheter (arrow) distally from the origin of PA in order to ensure targeted embolization of the prostate, which we can identify by the characteristic blush of contrast enhancement.

We can often identify PAs that have a common trunk with the superior or inferior ves-ical artery (Figure 7) that require the microcatheter to be placed after the origin of the vesical arteries in order to manage a targeted embolization.

#### 3.2.2. Anastomoses of PA with Penile Artery

**Figure 8:**
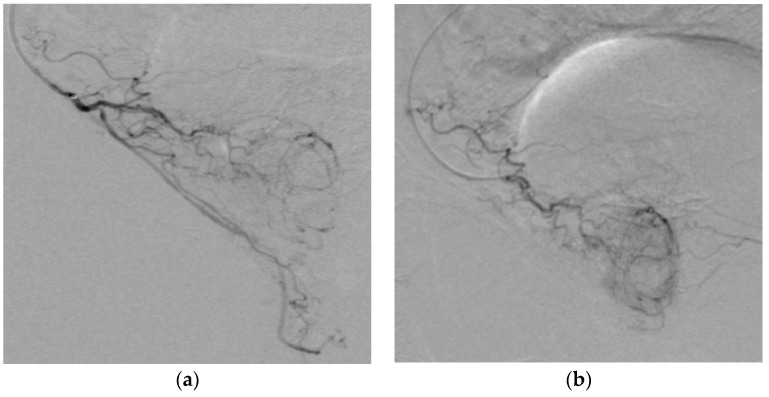
(**a**) Catheter positioned in proximal part of PA; we can identify arterial branches for the penis. Embolization should be made distally from the origin of these branches in order to have a targeted embolization. (**b**) Correct position of the microcatheter, distal from the origin of penile arterial branches, ensures targeted embolization of the prostate.

For arterial anastomosis and collaterals, we have to follow the same rule in order to obtain targeted embolization (Figure 8); otherwise, embolization particles will reach unwanted territories and may cause small areas of necrosis. What are most common and with severe symptoms are the bladder, penis, and rectum, causing urinary and sexual function deficiency and ischemic rectitis with inferior abdominal pain, respectively [2]. 

#### 3.2.3. Middle Rectal Artery with Origin from PA

**Figure 9:**
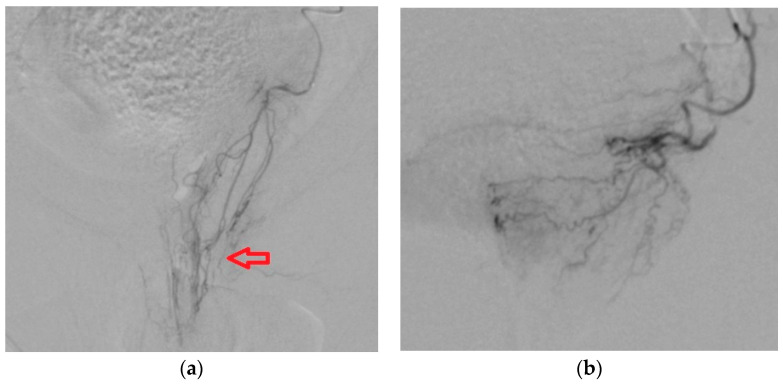
(**a**) Rectal arteries characteristic vertical blush (arrow). (**b**) Correct position of the microcatheter with the characteristic prostatic enhancement pattern.

We can find cases of middle rectal artery with the origin at the level of posterior PA, and as a key finding we can search for the characteristic vertical blush of the rectal arteries. In this case we have to correct the position of the microcatheter at the level of the anterior PA in order to perform the targeted embolization and not damage the rectal tissue [16]. Always before embolization, we have to demonstrate the correct position with DSA acquisition in which we can identify the prostatic blush (Figure 9).

### 3.3. Post-Procedural Short-Term Complications

We report no major periprocedural events for any of the patients included in this study such as non-targeted embolization with areas of necrosis at the level of the rectum, bladder, or penis. None of the patients included in this study had vascular complications after the procedure such as pseudoaneurysms or arterial dissection. Minor post-procedural side-effects have been reported such as pelvic pain in the first 24 h at 22 of the patients from the 35 included in this study (62.8%). 

## 4. Discussion

Because the PAE procedure has become increasingly popular in recent years, it is important to discuss in this paper the method and challenges encountered related to the anatomical variability of PA origin. The variability in the male pelvic arterial vascularization is significant; nevertheless, some patterns are identified as recurrent. Compared to the present literature, the results of this study agree with the scientific consensus that there are three most frequent origins, which are the IPA, anterior common trunk, and SVA. However, our paper, as well as the ones which we have cited, found different proportions of the main origins, which might be explained by regional differences. This is fundamental to improvement of the procedure, especially taking the exposure to radiation of both the patient and doctor into consideration. 

PAE is a demanding procedure and the outcome is directly related to the skills and the knowledge of the performing doctor, so it is necessary to highlight certain helpful information. The left brachial with a 5F introducer represents a better approach that will make the catheterization of both sides of the pelvis easier and also result in less frequent post-procedural local complications. 

The five aforementioned steps of arterial mapping before the embolization represent a coordinated search of the PA that will significantly reduce the time of the procedure and the radiation exposure to both the patient and the doctor, by having the typical anatomical variations in mind. When using the arterial mapping, we can correlate the results of the current study that will help identify the PA based on the most frequent or less frequent origins. The similar results of the current study compared to the cited studies represent a confirmation of the accuracy of our data.

The characteristic “cork-screw” aspect of the PA is helpful in identifying it in the numerous pelvic small vessels.

After the supra-selective catheterization of the PA, it is recommended to use 200–300 µm embolic particles highly diluted with contrast agent and saline solution injected slowly for the embolization. Repeated verification with DSA is needed in order to prevent embolization particle reflux from the PA in other arterial branches.

During the procedure, there are some challenges that can be encountered such as the angulation of the artery from the origin. The path and branches that may have an origin at this level are highly susceptible to variability and may represent a challenge for young interventional radiologists. The solution is represented by performing the end part of the microguide with a similar angle of the artery for a better navigation and catheterization. 

BPH is one of the most frequent pathologies in the elderly male population; at the same time, atherosclerotic disease is very common in this age group. In many cases of PAE, it is expected to encounter vascular stenosis and tortuosity that will transform the catheterization of the prostatic artery in the most difficult part of the procedure and increase the radiation exposure to the patient and doctor [17,18,19]. The position of the JR catheter is important and helpful in these cases because it can ensure the proper support for the microcatheter navigation through the atherosclerotic plaques. 

It is required to identify the collaterals or anastomosis of the PA before embolization in order to take proper action to ensure targeted embolization. The most frequent collateral arteries are with middle rectal artery, inferior vesical arteries, and IPA. If supra-selective catheterization is not possible, micro-coil embolization of the branch is recommended in order to ensure a proper embolization [6].

The endovascular navigation technique for the pelvic arterial supply discussed in this paper can find its application also in other special procedures that are intended to solve rare post-surgical complications such as bleeding and pseudoaneurysm that may occur. Cases with a very good outcome have been reported with a low morbidity [20,21]. It is essential to adapt the embolic material (coil, glue, particles) depending on the affected branch of the IIA.

Targeted embolization is one of the most important steps for a successful embolization procedure because it reduces the chances of post-procedural complications [22]. All aspects discussed must be taken into consideration when embolization is performed in order to have an efficient procedure without other post-procedural complications.

## 5. Conclusions

The PAE procedure is a safe and effective method of treatment for LUTSs in BPH as long as there is proper knowledge of the technique and anatomic variability of the arterial vascularization in the pelvic region. Due to its minimally invasive nature and low rate of complications, the popularity of this procedure has increased in the past years.

Compared to other studies, we noticed a difference in the percentage of the origins, but the most frequent variations are agreed upon in literature. It is important for young interventional radiologists to be aware of all the possible origins of PAs in order to be able to catheterize the PA and offer a proper treatment to their patients with BPH. 

## Figures and Tables

**Table 1:** Yamaki classification of internal iliac artery branching patterns.

Group	Branching Pattern
Group A	The internal iliac artery divides into two branches, the superior gluteal artery and the common trunk of the inferior gluteal and internal pudendal arteries.
Group B	The internal iliac artery divides into two branches, the internal pudendal artery and the common trunk of the superior gluteal and inferior gluteal arteries.
Group C	The internal iliac artery simultaneously divides into three major branches.
Group D	The internal iliac artery divides into the common trunk for the superior gluteal and internal pudendal arteries and the inferior gluteal artery.

**Table 2:** Comparison of recent studies results regarding the origin of PA.

Study	IPA (%)	Anterior Gluteal Trunk (%)	SVA (%)	Obturator Artery (%)	IGA (%)
Serbanoiu et al.	26 (37.1%)	19 (27.1%)	15 (21.4%)	8 (11.4%)	2 (2.8%)
Bilhim (2010) [14]	28 (56%)	14 (28%)	-	6 (12%)	2 (4%)
Bilhim (2012) [13]	73 (34%)	38 (17.8%)	42 (20%)	27 (12.6%)	8 (3.7%)
Zhang (2015) [7]	32 (27.9%)	45 (39.5%)	37 (32.6%)	-	-
DeAssis (2015) [11]	45 (31.1%)	-	43 (28.7%)	28 (18.8%)	-
Garcia-Monaco (2015) [15]	8 (17.4%)	26 (56.6%)	-	2 (4.3%)	-

## Data Availability

Data are available upon request from the corresponding authors.

## Abbreviations

BPH	benign prostatic hyperplasia
PAE	prostatic arterial embolization
DSA	digital subtraction angiography
PA	prostatic artery
SGA	superior gluteal artery
IGA	inferior gluteal artery
OBT	obturator artery
IIA	internal iliac artery
SVA	superior vesical artery
